# Attention-Deficit/Hyperactivity Disorder as a Mediating Variable for Invalid Baseline Profiles on the ImPACT

**DOI:** 10.3390/healthcare13131579

**Published:** 2025-07-01

**Authors:** Andre Petrossian, Louise A. Kelly, Rachel N. Casas, Jennifer M. Twyford, Michael A. McCrea, Thomas McAllister, Steven P. Broglio, Holly Benjamin, Thomas Buckley, Stefan Duma, Joshua Goldman, April Hoy, Jonathan Jackson, Thomas Kaminski, Christina Master, Christopher Miles, Nicholas Port, Adam Susmarski

**Affiliations:** 1 Graduate School of Psychology, California Lutheran University, Thousand Oaks, CA 91360, USArachelncasas@gmail.com (R.N.C.); jtwyford@callutheran.edu (J.M.T.); 2 Department of Exercise Science, California Lutheran University, Thousand Oaks, CA 91360, USA; 3 Departments of Neurosurgery and Neurology, Medical College of Wisconsin, Milwaukee, WI 53226, USA; mmccrea@mcw.edu; 4 Department of Psychiatry, Indiana University School of Medicine, Indianapolis, IN 46202, USA; twmcalli@iupui.edu; 5 Michigan Concussion Center, University of Michigan, Ann Arbor, MI 48109, USA; broglio@umich.edu; 6 Department of Rehabilitation Medicine and Pediatrics, University of Chicago, Chicago, IL 60637, USA; 7 Department of Kinesiology & Applied Physiology, University of Delaware, Newark, DE 19716, USAkaminski@udel.edu (T.K.); 8 Department of Biomedical Engineering and Mechanics, Virginia Tech University, Blacksburg, VA 24061, USA; 9 Department of Family Medicine and Orthopedics, University of California, Los Angeles, CA 90095, USA; jgoldman@mednet.ucla.edu; 10 Department of Athletics, School of Behavioral and Applied Sciences, Azusa Pacific University, Azusa, CA 91702, USA; areed@apu.edu; 11 Department of Family Medicine, United States Air Force Academy, Colorado Springs, CO 80840, USA; jonathan.c.jackson10.mil@mail.mil; 12 Division of Pediatric Orthopedics, Children’s Hospital of Philadelphia, Philadelphia, PA 19104, USA; master@chop.edu; 13 Department of Family and Community Medicine, Wake Forest University, Winston-Salem, NC 27109, USA; cmmiles@wakehealth.edu; 14 School of Optometry, Indiana University, Bloomington, IN 47405, USA; nport@indiana.edu; 15 Department of Orthopedics and Sports Medicine, United States Naval Academy, Annapolis, MD 21402, USA; susmarsk@gmail.com

**Keywords:** concussion, sports, assessment, ImPACT, ADHD, validity

## Abstract

Background: Individuals with ADHD may perform poorly on tasks targeting executive functioning skills such as the ImPACT, which requires the test-taker to employ judgement in non-routine situations Objective: To determine whether ADHD serves as a mediating variable for increasing the likelihood of an invalid score. Materials and Methods: A total of 39,140 collegiate athletes and United States military cadets consented to the Concussion Assessment, Research, and Education (CARE) Consortium study. Participants completed the CARE Baseline Packet which included various sections through which study participants provide self-report data, including demographic, personal, and family history sections. The personal history portion of the CARE Baseline Packet addressed the participant’s neurological history, including self-reported diagnosis of ADHD and history of traumatic brain injury. Variables utilized for the current study included age, gender, race, ethnicity, the participant’s primary college sport, use of mouthguards for athletes competing in sports requiring them, and the presence of an ADHD diagnosis. Participants responded to a question, inquiring if they had ever been diagnosed by a medical professional with ADHD, ultimately producing a dichotomous yes/no response. Results: We found that participants with ADHD were more likely to produce invalid baseline scores (*ß* = −0.884; *p* < 0.001). Similar results were found when controlling for sex, race, age, sport played, mouthguard use, and number of previous concussions (*ß* = −0.786; *p* < 0.001). Sex, race, sport played, and mouthguard use each played a significant role in determining profile validity, independent of ADHD diagnosis. With ADHD removed from the model, age negatively affected the likelihood of a valid score (*ß* = −0.052; *p* = 0.048). Conclusions: Our study suggests that the relationship between age and ImPACT validity is explained by the presence of ADHD. Results support adjusting ImPACT’s validity thresholds for individuals with ADHD.

## 1. Introduction

Concussion rates among National Collegiate Athletic Association (NCAA) athletes have increased since 1988 [1,2]. Defined as a mild brain injury caused by direct or indirect biomechanical forces transmitted to the head that results in a series of neuro-metabolic changes and temporary neurological dysfunction, the total number of sports-related concussions (SRCs) sustained in the United States is estimated to be between 1.6 and 3.8 million annually [3,4,5,6,7]. Concussion risk peaks between the ages of 9 and 22, coinciding with the popularity of group athletics [8].

Neuropsychological assessments are an essential component of concussion evaluation and management [9,10,11,12,13]. Comparing pre-injury baseline scores to those obtained following a suspected concussion plays an important role in both treatment and return-to-play decisions. The most widely used neurocognitive assessment in sports is Immediate Post-Concussion Assessment and Cognitive Testing (ImPACT) [14,15]. Developed to provide a computerized neuropsychological testing battery in an athletic environment, the instrument is broken down into three main parts: demographic data, neuropsychological tests, and the Post-Concussion Symptom Scales (PCSS). Together, the three sections work to provide data needed to assist in accurately diagnosing and managing head injuries [16,17]. ImPACT also contains a validity index specifically designed to aid in the identification of an invalid score. Common causes of invalid test profiles on the ImPACT include failure to properly read test directions; the presence of Attention-Deficit Disorder (ADD) and/or hyperactivity (ADHD); excessive fatigue; “horseplay”; left–right confusion; the presence of a neurological or psychological disorder in which impaired cognitive function is commonly present, and sandbagging, or intentional underperformance [18]. A survey of certified athletic trainers revealed, despite 94.7% of respondents administering baseline neurocognitive testing to their athletes, only 51.9% reported examining the scores for validity [19].

### 1.1. ADHD

Prevalence rates of college students diagnosed with ADHD in the United States vary greatly, with studies estimating between 0.5% and 5% meet criteria for the disorder [20]. Studies have linked individuals with ADHD to increased risk for bodily injury across the lifespan [21,22,23,24,25,26,27,28]. These risks may also have adverse effects when competing in sports. Adolescent and NCAA Division-I student athletes with ADHD both reported an increased number of concussions compared to peers, suggesting that traits associated with ADHD can place individuals at a general risk for increased injury [29,30,31].

ADHD has been linked to deficiencies in executive functioning [32,33,34,35]. This suggests that individuals with ADHD may perform poorly on tasks targeting executive functioning skills such as the ImPACT, which requires the test-taker to employ judgement in non-routine situations [36]. These factors support the need for additional research on concussion prevalence, symptomology, and recovery in athletes with premorbid ADHD diagnosis to ensure they are receiving the best care and return-to-play guidance.

### 1.2. The Present Study

We sought to explore the effect of ADHD on the validity of a participant’s baseline ImPACT profile. Utilizing validity indicators developed by the ImPACT, the goal of this study was to identify whether ADHD serves as mediating variable in increasing the likelihood of the participant triggering at least one of the invalidity indicators and producing an invalid score profile. A variable function as a mediator based on the level to which it accounts for the relationship between the independent and the dependent variables, particularly when the strength of the relationship between the independent and dependent variable is reduced by including the mediator [37,38]. The effect of additional statistical controls including sex, race, ethnicity, sport played, mouthguard use, age at the time of baseline testing, and the number of previous concussions on ImPACT validity were also explored.

Differences in baseline neuropsychological functioning, reported concussion symptoms, symptom presentation, diagnosis, and treatment have been identified between male and female athletes, athletes from various racial, ethnic, and socioeconomic backgrounds, and athletes who participate in different sports [19,39,40,41,42,43,44]. Older athletes have been found to outperform younger athletes on computerized neurocognitive testing measures and younger athletes have been found to produce an increased number of invalid ImPACT baseline scores compared to older athletes [45,46,47,48,49,50]. Mouthguard use has been a debated topic, with research both supporting and refuting their role in reducing risk of concussion [51,52,53,54,55]. Studies have also identified links between an ADHD diagnosis and a general risk for increased injury [21,27,28,30,31]. Symptoms of ADHD including difficulties with concentration and attention may result in less adherence to mouthguard use by athletes with ADHD, potentially increasing injury risk to an already vulnerable population. Finally, studies have explored the potential link between concussion history and baseline concussion test performance, albeit with variable results [56,57,58,59]. We hypothesized that the presence of ADHD in a group of collegiate athletes and U.S. Military cadets will serve as a mediating variable for increasing the probability of producing an invalid baseline profile score on the ImPACT.

## 2. Materials and Methods

Data utilized in the current study were obtained for secondary analysis from the National Collegiate Athletic Association (NCAA) and Department of Defense Concussion Assessment, Research, and Education (CARE) Consortium. The Consortium gathers a variety of information from student athletes at member institutions including baseline and post-injury concussion test data in addition to detailed demographic information. Prior to the start of each season, athletes complete a battery of assessments targeting concussion-related symptoms, a measure of cognitive abilities, and evaluations of postural stability which serve as their baseline level of performance. Initial baseline assessments took between 55 and 60 min to complete and approximately 45 min to complete each successive year. A more in-depth overview of the CARE Consortium methodology can be found elsewhere [60,61]. Consent for participation in the longitudinal study was obtained through written informed consent by all eligible participants following a protocol approved by both the institution’s local institutional review board (IRB) as well as the United States Army’s Human Research Protection Office.

### 2.1. The CARE Baseline Packet

The demographic portion of the CARE Baseline Packet includes various sections through which study participants provide self-report data, including demographic, personal, and family history sections. The personal history portion of the CARE Baseline Packet addressed the participant’s neurological history, including self-reported diagnosis of ADHD and history of traumatic brain injury. Variables utilized for the current study included age, gender, race, ethnicity, the participant’s primary college sport, use of mouthguards for athletes competing in sports requiring them, and the presence of an ADHD diagnosis. Participants responded to a question, inquiring if they had ever been diagnosed by a medical professional with ADHD, ultimately producing a dichotomous yes/no response. Located in the CARE baseline packet under Medical History, each participant was asked to respond in a dichotomous yes/no fashion to the following question: “Have you ever been diagnosed by a Physician/MD with Attention Deficit-Hyperactivity Disorder (ADD/ADHD)?” (NCAA-DoD Grand Alliance Baseline Packet v.6, p.7). The participant’s response to this question served as the focal independent variable for the present study.

### 2.2. Immediate Post-Concussion Assessment and Cognitive Testing (ImPACT)

Categorized as a Level A neurocognitive functioning measure in the original study, the ImPACT was utilized by 25 of 30 sites (see Table 1) [60,61]. A 25-min computerized test, the ImPACT generates composite scores that quantify performance in domains such as attention span, working memory, sustained and selective attention time, non-verbal problem solving, and reaction time [18]. Analysis and comparison of scores on the three sections provide data needed to assist in the diagnosis and management of head injuries [16,17]. The ImPACT contains a validity index that uses a specific algorithm to flag invalid scores. The ImPACT manual states that scores are deemed invalid if one or more of the five validity indicators are failed. While failure of a single criterion may generate an invalid result, our data were limited to an overall binary validity indicator.

The following scenarios are identified as grounds for an invalid baseline examination. When any of these criteria are met, a sentence will appear in the score report stating that there may be some concern with the validity of the test results [18].

− X’s and O’s Total Incorrect > 30,
− Impulse Control Composite > 30,
− Word Memory Learning Percent Correct < 69%,
− Design Memory Learning Percent Correct < 50%, and
− Three Letters Total Letters Correct < 8%.

### 2.3. Research Design and Data Analysis

A binary logistic regression was chosen to analyze the obtained data due to the binary nature of the dependent variable, ImPACT profile validity [38,62,63]. Additional independent variables including ethnicity, race, gender, age, sport played, mouthguard use, and concussion history were applied to the regression model to explore their effects on the dependent variable both with and without the presence of an ADHD diagnosis. Additionally, the effects of these variables on ADHD itself were calculated to explore the potential of ADHD acting as a mediating variable on ImPACT validity.

### 2.4. Data Preparation

Statistical analysis was conducted on IBM SPSS Statistics software version 26. The data set was restricted to cases with complete data on the studies’ dependent variable of ImPACT score validity. This reduced the data set from the original 47,397 cases to 39,140 cases, representing a 17.4% attrition of cases. The remaining sample included 25,172 males (64.3%) and 13,968 females (35.7%) with a mean age of 19.44 years (SD = 1.44). Of the remaining participants, 1758 (4.6%) self-reported having an ADHD diagnosis and 672 (1.7%) produced an invalid ImPACT baseline profile. (See Table 2 for a complete list of percentages and frequencies of study variables.)

The CARE baseline packet asked participants to identify both their race and ethnicity. Given the fact that a respondent’s racial classification was a multi-category nominal-level variable, a series of seven dummy variables were created for use within the various regression equations. The categories that were retained as dummy variables in all regression equations for race classification included African American, Asian, Hawaiian or Pacific Islander, Indian or Alaskan Native, White, and Multiple Races. Hispanic/Latino or Not Hispanic/Latino were retained as ethnicity classifications.

### 2.5. Participants

Participants identified their race as African American (*n* = 4531, 11.6%); American Indian/Alaskan Native (*n* = 164, 0.4%); Asian (*n* = 1708, 4.4%); Native Hawaiian/Pacific Islander (*n* = 260, 0.7%); White (*n* = 3003, 7.7%); or Multiple Races (*n* = 3003, 7.7%). Participant ethnicity statistics included those who identified as Hispanic/Latino (*n* = 3708, 9.5%) or Not Hispanic/Latino (*n* = 35,432, 90.5%).

Participants were also asked to identify their primary college sport, with sports utilized in the current study including those with over 500 participants. The number of participants for each sport retained in the current study were as follows: baseball (*n* = 1637, 4.2%); basketball (*n* = 1539, 3.9%); football (*n* = 5030, 12.9%), ice hockey (*n* = 655, 1.7%), lacrosse (*n* = 1144, 2.9%); soccer (*n* = 2658, 6.8%); and softball (*n* = 870, 2.2). (See Table 2 and Table 3 for a complete list of percentages, frequencies, means, and standard deviations of study variables.)

## 3. Results

ImPACT profile validity was established as the dependent variable with ADHD as the mediator variable. Tabular results in support of this model are presented in Table 4. Four models were devised, and independent variables were entered manually into SPSS in a hierarchical fashion. Model 1 explored the direct effect of ADHD on ImPACT profile validity, while Model 2 explored the direct effect of the statistical controls including sex, race, ethnicity, sport played, mouthguard use, age at the time of baseline testing, and the number of previous concussions on ImPACT profile validity when ADHD was not included in the model. Model 3 explored the effect of these same statistical control variables on ImPACT validity when controlling for ADHD. Finally, Model 4 explored the direct effects of the statistical control variables on ADHD.

Based on the application of a binomial logistic regression, ADHD was found to have a direct effect on ImPACT score validity individually (*ß* = −0.884; *p* < 0.001), representing a 57.0% reduction in the proportional odds of the ImPACT profile being valid (Table 4, Model 1). When controlling for the statistical covariates of sex, race, age, sport, mouthguard use, and the number of concussions, the presence of a self-reported ADHD diagnosis also had a direct effect on the validity of ImPACT scores (*ß* = −0.786; *p* < 0.001), with a 54.4% reduction in the proportional odds of the ImPACT test being valid (Table 4, Model 3).

Several of the statistical control variables influenced ImPACT validity when controlling for ADHD including the sex of the respondent and the respondent’s identified race. Being male reduced the odds of the ImPACT score being valid (*ß* = −0.189; *p* = 0.049), while identifying as White increased the odds of the ImPACT being valid (*ß* = 0.639; *p* = 0.001. The type of sport played by respondents also influenced ImPACT validity when controlling for ADHD. Specifically, playing football or lacrosse lowered the odds of the ImPACT being valid (*ß* = −0.589; *p* < 0.001; *ß* = −0.593; *p* = 0.014), while playing soccer increased the odds of the ImPACT profile being valid (*ß* = 0.386; *p* = 0.045). Athletes who reported using a mouthguard were shown to have increased odds of producing valid ImPACT scores when controlling for ADHD (ß = 0.353; *p* = 0.003).

When ADHD was not included in the model, age at baseline lowered the odds of the ImPACT being valid (ß = −0.052; *p* = 0.048). This result suggests that the direct effect of age on ImPACT validity is mediated by the presence of ADHD in the model (Table 4, Model 2). Finally, several statistical controls, including the respondent’s sex (ß = −0.153; *p* = 0.010), identifying as Asian (ß = −0.811; *p* = 0.003), age at baseline (ß = 0.080; *p* < 0.001), mouthguard use (ß = −0.464; *p* < 0.001), number of previous concussions (ß = 0.271; *p* < 0.001), and playing every sport other than Softball all had direct effects on ADHD. These direct effects suggest that ADHD functions as a mediator variable (Table 4, Model 4)

## 4. Discussion

The current study explored the mediating effect of ADHD on the validity of baseline profile scores on the ImPACT. Although previous studies have identified a connection between a diagnosis of ADHD and invalid scores on the ImPACT, the current study is one of, if not the first, to identify these results in such a large and diverse sample. To the author’s knowledge, this research represents the first study that uses statistical methods to treat ADHD as a mediating variable in this particular setting. The results carry actionable clinical implications: Their findings stress the importance of creating distinct norms for ADHD or revised validity thresholds to support accurate baseline assessment for individuals with ADHD.

The interpretation of the mediation model becomes more robust with the inclusion of effect size estimates. Model 1 revealed a 57.0% decrease in the proportional odds of producing a valid ImPACT score among ADHD participants with an odds ratio of 0.43 that decreased to a 54.4% reduction with an odds ratio of 0.46 after covariates were introduced in Model 3. The provided odds ratios help in understanding the practical meaning behind the observed relationships.

It is important to us to avoid unintentional reinforcement of harmful stereotypes. Our analysis purposefully avoids labeling athletes in football and lacrosse as pathological despite findings showing lower odds of valid scores for these groups. These findings are interpreted through the perspective of high-collision sport demands and equipment use alongside executive function requirements while maintaining awareness of potential biases. Our study included an analysis to determine if there exists any correlation between mouthguard usage in contact sports and the ADHD status. The analysis presented in Table 4, Model 4 demonstrates that mouthguard use shows a significant negative association with ADHD diagnosis (β = −0.464, *p* < 0.001), indicating that individuals with ADHD are less likely to wear mouthguards. Mouthguard use merits additional research because it could function as an observable indicator of executive function or compliance behavior.

Given these findings, future research should explore the intersection of executive function, compliance behaviors (e.g., equipment adherence), and concussion outcomes in neurodiverse athletes. We advise scientific peers to handle demographic covariates carefully and empathetically while we suggest additional research into the impact of environmental and systemic variables on neurocognitive assessment performance in different racial and athletic categories.

The current study explored the mediating effect of ADHD on the validity of baseline profile scores on the ImPACT. Although previous studies have identified a connection between a diagnosis of ADHD and invalid scores on the ImPACT, the current study is one of, if not the first, to identify these results in such a robust sample size. To the author’s knowledge, it is also the first study to identify the mediating effects of ADHD on the score validity of baseline ImPACT profiles. The current study has several important implications relevant to baseline testing procedures and normative data collection. First and foremost, the results give merit to the development of modified validity cutoffs for individuals with a confirmed ADHD diagnosis. An alternative solution may involve the establishment of normative data sets that would provide more accurate validity indicators for groups of athletes such as those diagnosed with ADHD.

Four out of the five ImPACT validity indicators listed in the current manual evaluate components of executive functioning. Given the link to ADHD and executive functioning, one could argue athletes with ADHD are at a potential disadvantage for producing valid scores with the current validity indicators. Subsequently, these athletes may be required to retake the baseline assessment later, even though they may produce a similarly invalid result. An invalid baseline test may result in a lack of personalized baseline data for the athlete in question. This may have an adverse effect on concussion management and treatment, likely complicating return-to-play decisions as team medical staff may be forced to rely on self-report data to make return-to-play decisions. Utilizing such as approach may result in the premature resumption of physical activity following a concussion, potentially increasing the risk of additional injury, and compromising the neurological health of the athlete.

Numerous studies have identified links between an ADHD diagnosis and a general risk for increased injury, including head trauma [21,22,23,24,25,26,27,28,30,31]. Additional research identifying ADHD as a risk factor for concussions in a sample of Division-I college athletes supports the need to ensure athletes with ADHD are afforded the opportunity to produce valid baseline ImPACT scores [29]. This notion is further supported by research that found athletes with ADHD not only have a higher prevalence of self-reported concussions but also respond differently to normal controls on concussion assessment measures [64]. Modified validity cutoffs or the establishment of normative data sets for individuals with ADHD may also help differentiate between invalid scores resulting from neurological conditions and those due to intentional underperformance.

This study is not without limitations. First and foremost, all athletes self-reported their ADHD diagnosis. Although self-reported medical history is widely used and has shown some validity in other studies, this can potentially limit the generalizability of the findings due to subjectivity, memory errors, intentionally underreporting, or overreporting of symptoms by participants, thus highlighting the need for future studies using validated diagnostic criteria and performance validly measures to parse apart genuine impairment from potential malingering [29]. While our findings suggest a mediating effect of ADHD on ImPACT validity, the accuracy of ADHD self-reports cannot be verified, and intentional underperformance (sandbagging) remains a plausible alternative explanation. Furthermore, the language in the CARE Baseline Packet specifically refers to ADHD diagnosed by a physician or MD; potentially excluding ADHD diagnosed by mental health providers. Participants who self-reported an ADHD diagnosis may currently, or may have in the past, received medications or applied behavioral interventions to manage their symptoms, thus the true effect of ADHD on ImPACT score validity may be higher than reported. Additional limitations including environmental and co-morbid psychological factors may have also affected score validity.

Results of the current study confirm the hypothesis that ADHD serves as a mediating variable in producing invalid baseline profiles on the ImPACT. In a large sample of collegiate athletes and United States Military cadets, the presence of ADHD affected the relationship between the statistical controls and the dependent variable of ImPACT profile validity. When controlling for ADHD, sex, race, sport played, and mouthguard use each played a significant role in determining ImPACT profile validity. This confirmed the relationship between the independent and dependent variables, thus establishing the foundation for mediation. A respondent’s sex, being of Asian descent, reported younger age at baseline, playing every sport other than softball, mouthguard use, and the number of previous concussions all played a significant role in determining the presence of an ADHD diagnosis. Although literature suggests that Asian students are generally underdiagnosed with ADHD, our regression results identified that Asian students in this sample were statistically less likely to report an ADHD diagnosis (ß = −0.811, *p* = 0.003). The confusion likely arose from the complexity of the statistical mediation model. We have clarified in the revised discussion that while Asian ethnicity was significantly negatively associated with ADHD, their performance on the ImPACT was still analyzed alongside other covariates. These direct effects provide additional support that ADHD may function as a mediator variable, as identifying a relationship between the independent variables and the mediator variable constitutes the first stage in Baron and Kenny’s (1986) mediation analysis [37]. ADHD negatively affected score validity when controlling for covariates of sex, race, age, sport played, mouthguard use, and the number of previous concussions, albeit to a slightly lesser extent. This established Baron and Kenny’s (1986) second stage of mediation analysis which states the mediator variable (ADHD) should significantly relate to the dependent variable (ImPACT validity) [37]. When ADHD was not included in the model, the participant’s age at baseline lowered the odds of the ImPACT score being valid. This suggests the relationship between age and ImPACT validity is explained by the presence of an ADHD diagnosis, thus satisfying the final condition of Baron and Kenny’s (1986) model and confirming ADHD functions as a mediator variable [37].

## 5. Conclusions

These findings validate the need for additional research on adjusting the ImPACT’s validity thresholds for individuals diagnosed with ADHD. Such efforts will ensure the most accurate, inclusive methods of baseline concussion assessment are employed to aid in accurate concussion diagnosis and informed return-to-play decisions. Modification to the ImPACT’s validity indicators may aid in providing further diagnostic clarity for medical professionals evaluating return-to-play decisions for athletes diagnosed with ADHD. This shift towards a more inclusive method of determining ImPACT score validity will make a positive ImPACT in protecting the neurological health of all athletes, including those with executive functioning challenges.

## Figures and Tables

**Table 1:** Level A Assessment Measures.

Neurocognitive Screening Measures
Standard Assessment of Concussion (SAC)
Sport Concussion Assessment Tool 3 (SCAT 3)
Brief Symptom Inventory 18 (BSI-18)
Neurocognitive Functioning Measures
Computerized Neurocognitive Software Vital Signs
Cogstate Computerized Assessment Tool (Cogstate CCAT)
Immediate Post-Concussion Assessment and Cognitive Testing (ImPACT)

**Table 2:** Percentages and Frequencies of Study Variables.

	Frequency	Percent
Sex:		
Female	13,968	35.7%
Male	25,172	64.3%
Race: African American	4531	11.6%
Race: Asian	1708	4.4%
Race: Hawaiian or Pacific Islander	260	0.7%
Race: Indian or Alaskan Native	164	0.4%
Race: Multiple Races	3003	7.7%
Race: White	29,474	75.3%
Ethnicity: Hispanic	3708	9.5%
Sport: Baseball	1637	4.2%
Hockey	655	1.7%
Lacrosse	1144	2.9%
Soccer	2658	6.8%
Softball	870	2.2%
Basketball	1539	3.9%
Football	5030	12.9%
Mouthguard used?		
Other than yes	27,655	70.7%
Yes	11,485	29.3%
Diagnosed w/ADHD		
Other than yes	37,382	95.5%
Yes	1758	4.5%
ImPACT test results valid? (dependent variable)		
No	672	1.7%
Yes	38,468	98.3%
*N*	39,140	100.0%

**Table 3:** Binary Logistic Regression of ImPACT Test Results on the Independent Predictors.

	Model 1			Model 2			Model 3		
Variable	ß	exp (ß)	*p*	ß	exp (ß)	*p*	ß	exp (ß)	*p*
Constant	4.104	60.585	0.000	4.744	114.887	0.000	4.684	108.225	0.000
Q34. Diagnosed w/ADHD	−0.844	0.430	0.000				−0.786	0.456	0.000
Q11. Sex				−0.189	0.828	0.049	−0.197	0.821	0.041
Q12. Race: African American				−0.003	0.997	0.990	0.015	1.016	0.943
Q13. Race: Asian				0.235	1.265	0.372	0.222	1.249	0.399
Q14. Race: Hawaiian or Pacific Islander				−0.333	0.716	0.359	−0.339	0.712	0.351
Q15. Race: Indian or Alaskan Native				0.851	2.342	0.247	0.857	2.356	0.244
Q16. Race: Multiple Races				0.298	1.348	0.201	0.311	1.365	0.183
Race: White 29,474 75.3%				0.639	1.895	0.001	0.655	1.926	0.001
Q21. Ethnicity: Hispanic				−0.104	0.901	0.441	−0.108	0.898	0.426
Q29. Age at baseline				−0.052	0.949	0.048	−0.047	0.954	0.072
Q30_1: Baseball				0.071	1.073	0.734	0.118	1.125	0.573
Q30_2: Hockey				0.289	1.335	0.489	0.312	1.366	0.456
Q30_3: Lacrosse				−0.593	0.552	0.014	−0.536	0.585	0.028
Q30_4: Soccer				0.386	1.471	0.045	0.400	1.491	0.038
Q30_5: Softball				−0.056	0.946	0.848	−0.049	0.952	0.866
Q30_6: Basketball				0.074	1.077	0.713	0.090	1.095	0.655
Q30_7: Football				−0.589	0.555	0.000	−0.542	0.582	0.000
Q31. Mouthguard used?				0.353	1.424	0.003	0.337	1.400	0.005
Q33. How many previous concussions?				−0.078	0.925	0.164	−0.061	0.941	0.277
*N*	39,140			39,140			39,140		
Chi-square goodness of fit	32.752		0.000	110.610		0.000	138.857		0.000
*df*	1			18			19		
Nagelkerke R^2^	0.005			0.018			0.022		

**Table 4:** Binary Logistic Regression of ADHD on the Independent Predictors.

	Model 4		
Variable	*ß*	*exp* (*ß*)	*p*
Constant	−4.999	0.007	0.000
Q11. Sex	−0.153	0.858	0.010
Q12. Race: African American	0.317	1.373	0.081
Q13. Race: Asian	−0.811	0.444	0.003
Q14. Race: Hawaiian or Pacific Islander	−0.202	0.817	0.595
Q15. Race: Indian or Alaskan Native	0.180	1.197	0.670
Q16. Race: Multiple Races	0.184	1.202	0.334
Q19. Race: White	0.245	1.278	0.149
Q21. Ethnicity: Hispanic	−0.058	0.943	0.524
Q29. Age at baseline	0.080	1.083	0.000
Q30_1: Baseball	0.797	2.219	0.000
Q30_2: Hockey	0.549	1.732	0.002
Q30_3: Lacrosse	1.196	3.306	0.000
Q30_4: Soccer	0.284	1.328	0.003
Q30_5: Softball	0.133	1.143	0.430
Q30_6: Basketball	0.337	1.401	0.005
Q30_7: Football	1.038	2.823	0.000
Q31. Mouthguard used?	−0.464	0.629	0.000
Q33. How many previous concussions?	0.271	1.311	0.000
*N*	39,140		
Chi-square goodness of fit	393.653		0.000
*df*	18		
Nagelkerke R^2^	0.033		

## Data Availability

De-identified CARE data can be accessed via the Federal Interagency Traumatic Brain Injury Research database https://fitbir.nih.gov (accessed on 26 June 2025).

## Abbreviations

The following abbreviations are used in this manuscript:

NCAA	National Collegiate Athletic Association
SRC	sports-related concussions
ImPACT	Immediate Post-Concussion Assessment and Cognitive Testing
PCSS	Post-Concussion Symptom Scales
ADD	Attention-Deficit Disorder
ADHD	Attention-Deficit/Hyperactivity Disorder
CARE	Concussion Assessment, Research, and Education
IRB	institution’s local institutional review board
DOD	Department of Defense
